# A small deletion in *SERPINC1* causes type I antithrombin deficiency by promoting endoplasmic reticulum stress

**DOI:** 10.18632/oncotarget.12349

**Published:** 2016-09-30

**Authors:** Jingjing Su, Liang Shu, Zhou Zhang, Lei Cai, Xin Zhang, Yu Zhai, Jianren Liu

**Affiliations:** ^1^ Department of Neurology, Shanghai Ninth People's Hospital, Shanghai Jiao Tong University School of Medicine, Shanghai 200011, China; ^2^ Institute of Biliary Tract Disease Research, Shanghai Jiao Tong University School of Medicine, Shanghai 200092, China; ^3^ Bio-X Institutes, Key Laboratory for the Genetics of Developmental and Neuropsychiatric Disorders (Ministry of Education), Shanghai Key Laboratory of Psychotic Disorders (No.13dz2260500), Shanghai Jiao Tong University, Shanghai 200240, China; ^4^ Clinical Research Center of Shanghai Jiao Tong University School of Medicine, Shanghai 200092, China

**Keywords:** SERPINC1, deletion mutation, antithrombin deficiency, endoplasmic reticulum stress

## Abstract

Antithrombin (AT) deficiency is an autosomal dominant disorder, and identification of mutation AT variants would improve our understanding of the anticoagulant function of this serine protease inhibitor (SERPIN) and the molecular pathways underlying this disorder. In the present study, we performed whole-exome sequencing of a Chinese family with deep vein thrombosis, and identified a new small deletion that eliminates four amino acids (INEL) from exon 4 of SERPINC1 gene. This causes type I AT deficiency by enhancing the intracellular retention of this protein. AT retention leads to endoplasmic reticulum (ER) stress, which further inhibits AT release. In addition, ER stress activates ER-associated degradation, which promotes AT degradation. Suppression of ER stress enhanced the secretion of AT, while inhibition of ER-associated degradation suppressed AT release. Thus, our study identified a new mutation (INEL deletion) causing type I AT deficiency, and uncovered a novel mechanism for AT retention through enhanced ER stress, which may provide an innovative approach for treating AT deficiency.

## INTRODUCTION

Hemostasis and thrombosis are in an elaborate equilibrium, which is essential for animals and humans survival [1, 2]. During this physiological process, antithrombin (AT) is important for promoting anticoagulation (hemostasis) through various mechanisms – for instance, by inhibiting procoagulants such as Factor IXa, Factor Xa and thrombin [3, 4]. Accordingly, AT deficiency contributes substantially to thrombosis, and complete deficiency is fatal. As a member of the serine protease inhibitor (SERPIN) superfamily, AT is mainly synthesized in the liver and released into plasma [5, 6]. The human AT gene *SERPINC1* is located on chromosome 1q23.1-23.9, is 13.5 kb in length, and encodes a precursor of 464 amino acids, which is released into the plasma as a mature inhibitor after a 32-amino-acid peptide is cleaved off [7].

AT deficiency contributes to diverse thrombosis disorders, such as venous thrombosis [8]. There are two types of AT deficiency: quantitative defects (type I) due to reduced protein production, and qualitative defects (type II) due to abnormal structure and function [9]. Among patients with AT deficiency, the majority have a single mutation in *SERPINC1*, resulting in an AT level less than half of the normal level. Multiple mutations in *SERPINC1* are rare and could present themselves as either substantially reduced or undetectable AT activity [10].

AT deficiency is an autosomal dominant disorder, and identification of mutation variants of AT has helped to clarify the anticoagulant function of the SERPIN family [9]. Studies on variants have enabled the identification of the proteinase binding site, which is essential for heparin activation, and have provided valuable insights into the structure-function relationship [5]. Thus, the identification of new variants will undoubtedly provide further insight into the function and regulation of AT and increase the potential to treat AT deficiency. Thus far, more than 270 different mutations causing AT deficiency have been reported [11]. Most of the mutations are single mutations, minor deletions, or insertions, while deletions of long fragments are much less common [5, 10]. The most common clinical manifestations are pulmonary embolism and deep vein thrombosis (DVT), while cerebral artery or vein thrombosis is more rare [12].

The endoplasmic reticulum (ER) is the factory for protein biosynthesis and assembly. During these dynamic processes, misfolded and unfolded proteins produced in the ER, especially secreted and transmembrane proteins, instigate the evolutionarily conserved unfolded protein response and lead to ER stress [13, 14], which is fundamental for various pathological events, such as inflammation, aging, and neurodegenerative disorders [15, 16]. Multiple factors can cause ER stress, including mutations, altered cellular metabolism and infection, usually with the involvement of the ‘master sensor’ chaperone GRP78 (also known as BiP). Binding of misfolded proteins to GRP78 releases its inhibitory effect on inositol-requiring kinase 1 (IRE1), double-stranded RNA-activated protein kinase (PKR)-like endoplasmic reticulum kinase (PERK), and ATF6, and allows the initiation of the downstream transcriptional effectors XBP1s, ATF4, and pATF6-N, respectively, thus activating ER-associated degradation (ERAD) [16–19].

In this study, we performed whole-exome sequencing of a Chinese family with DVT, and identified a new small deletion causing the elimination of four amino acids (INEL), which leads to type I AT deficiency by enhancing the intracellular retention of AT. This retention causes ER stress, which promotes the proteasomal degradation of AT. Inhibition of ER stress enhanced the secretion of AT. Thus, our study potentially provides a novel way to treat AT deficiency.

## RESULTS

### Identification and clinical characteristics of patients with *SERPINC1* deletion mutation

**Figure 1:**
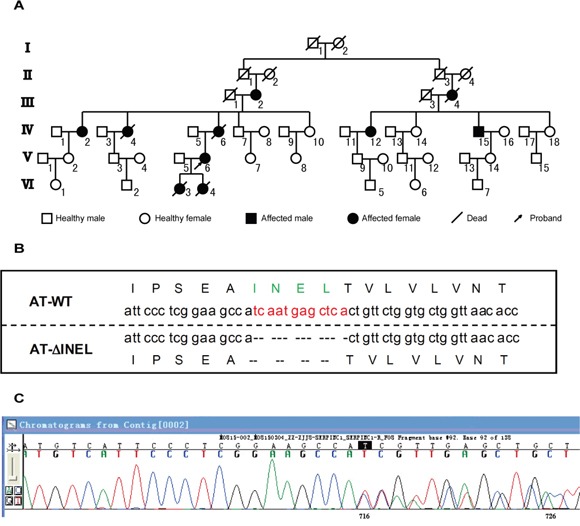
Identification of AT deficiency due to a small deletion **A.** Pedigree of proband. The proband is indicated by the arrow. Family members affected with thrombosis are indicated in black, and III-2, IV-2/12/15, and V-6 are the small deletion carriers. **B.** Representation of the residues deleted (ΔINEL) in the mutant AT aligned with the wild-type (WT) sequence. **C.** The sequencing graph for mutant AT.

We became aware of an AT-deficient family because one member (proband, V-6) came to our outpatient department and was found to have DVT and a cerebral ischemic attack (Figure 1A). We subsequently investigated her family members and found that 10 members (of 50 across six generations), five of whom were still living, had experienced venous thrombosis events. Her mother (IV-6) had experienced thrombotic events and died from cerebral venous sinus thrombosis at the age of 55, while there were no thrombotic events reported for her father and other siblings. However, one of her daughters (VI-3) had died from a “cerebral hemorrhage” at 1 day old, and another daughter (VI-4) had died for unknown reasons a couple of days after birth.

In order to elucidate new molecules involved in this disorder, we performed whole-exome sequencing of the proband (V-6), two other family members who had undergone thrombotic events (IV-12/15), and one normal control (IV-14), and discovered a deletion mutation in the *SERPINC1* gene (g.173879926GTGAGCTCATTGA>G) (Figure 1B, 1C), which eliminated the amino acid sequence INEL (239-242) from the gene product, AT (AT^ΔINEL^). As AT deficiency is usually inherited as an autosomal dominant disorder, we then analyzed plasma AT in the three patients (IV-12/15, V-6), and found remarkably low circulating AT antigen levels and activity, suggesting that these patients had type I AT deficiency (Table 1). Thus, we identified a new *SERPINC1* deletion leading to type I AT deficiency.

**Table 1:** Plasma AT antigen level and activity for patients

	Normal range	Patient 1	Patient 2	Patient 3
AT antigen level (mg/dL)	25-36	9.37	11.20	9.80
AT activity (%)	84.6-120.2	27	40	30

AT, antithrombin

### The accumulation of mutant AT activates ER stress

**Figure 2:**
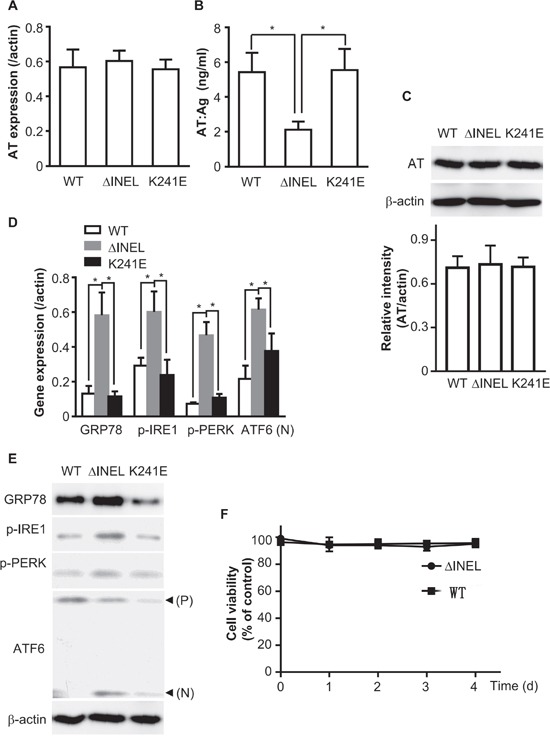
The accumulation of mutant AT activates ER stress HEK293 or LO2 cells were transiently transfected with ATWT, ATΔINEL or ATK241E plasmids for 48 hours. **A.** HEK293 cells were transiently transfected with the indicated plasmids. Total RNA was extracted and reverse-transcribed, and PCR performed with *AT*-specific primer pairs. **B.** LO2 cells were transiently transfected with the indicated plasmids, and culture medium was collected for ELISA detection of AT levels. **C.** LO2 cells were transiently transfected with the indicated plasmids, and cell lysates were analyzed by immunoblotting with AT antibodies. The band density was quantified with ImageJ software. The quantitative data were obtained from three independent experiments, and are shown as the mean ± SEM. **D.** LO2 cells were transiently transfected with the indicated plasmids. Total RNA was extracted and reverse-transcribed, and PCR was performed with the indicated specific primer pairs. **E.** Cell lysates were analyzed by immunoblotting with antibodies for the expression of the indicated molecules. The image is representative of three experiments. **F.** LO2 cells were plated in 96-well plates at a density of 5×10^2^ cells per well and transfected with a plasmid containing WT or mutant *AT*. The cell viability was assessed at various time-periods (0, 1, 2, 3, 4 d) after transfection. The absorbance of each well was measured at 570 nm with an ELISA reader. The cell viability was calculated as the percentage of the absorbance with respect to the control (WT). All the results are the mean ± SEM of three independent experiments (**A-F**). (*p<0.05)

Various mechanisms, such as RNA instability, impaired translation, protein misfolding and abnormal conformations, lead to intracellular retention of AT and thus contribute to type I AT deficiency. In order to further clarify the underlying mechanism for AT deficiency due to the newly discovered *SERPINC1* deletion (AT^ΔINEL^), we generated Myc-tagged full-length and mutant versions (AT^ΔINEL^ and AT^K241E^) of *SERPINC1*. K241E has been reported as another AT mutant (AT^K241E^) and served as a control here [20]. The full-length and mutated plasmids were used to transfect HEK293 cells, and real-time PCR was performed to investigate *AT* mRNA expression. *AT* mRNA levels were similar among the groups (Figure 2A), suggesting that INEL deletion causes AT deficiency posttranslationally.

As AT is mainly synthesized in hepatocytes [21], we also expressed the AT plasmids in the LO2 cell line (normal human fetal liver cells) by transient transfection. Cultured medium was collected and subjected to ELISA for AT. AT^ΔINEL^ LO2 cells secreted much less AT than WT or AT^K241E^ LO2 cells (Figure 2B), while there was no observable difference in AT levels among the groups as detected by Western blotting of cell lysates (Figure 2C), implying that AT^ΔINEL^ was retained intracellularly.

The ER is the production and folding workshop for secreted and transmembrane proteins, and misfolded proteins cause ER stress [16]. Thus, we hypothesized that AT retention induced ER stress. The heat shock protein GRP78 is the most abundant ER chaperone and is considered to be a key factor in the initiation of the unfolded protein response (UPR) [17], which activates ER stress sensors such as IRE1, PERK, and ATF6. We prepared total RNA from LO2 cells and determined the expression of ER stress-related molecules with a quantitative reverse transcriptase polymerase chain reaction (qPCR). AT^ΔINEL^ deletion significantly enhanced the RNA expression of GRP78, p-IRE1, p-PERK and ATF6 (Figure 2D). As expected, increased protein expression of GRP78, p-IRE1 and p-PERK, as well as cleavage of ATF6, were detected in AT^ΔINEL^ cells by Western blotting (Figure 2E). These data demonstrate that this mutation of AT causes cytoplasmic retention of AT and thus induces ER stress.

We also detected cell viability after transfecting cells with plasmids containing wild-type (WT) or mutant AT for various time-periods (0, 1, 2, 3, 4 d). There was no obvious change in cell viability during the various time periods, suggesting that persistent ER stress did not cause cell death (Figure 2F).

### Enhanced ER stress contributes to AT deficiency by promoting AT degradation

**Figure 3:**
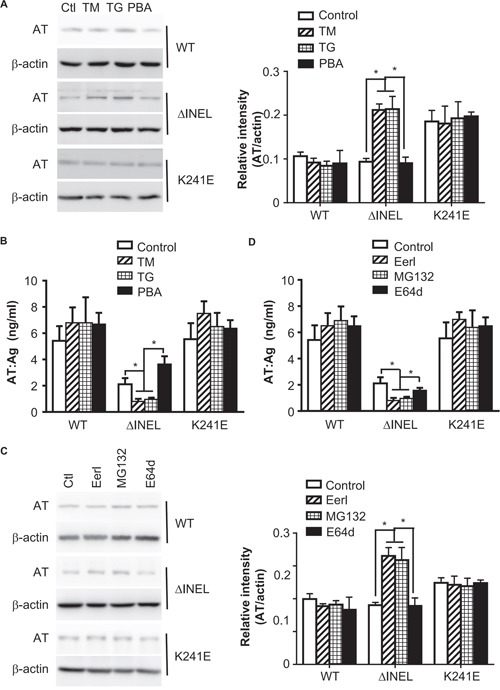
Enhanced ER stress contributes to AT deficiency by promoting AT degradation **A, B.** LO2 cells were transiently transfected with AT^WT^, AT^ΔINEL^ and AT^K241E^ plasmids for 48 hours, and then treated with tunicamycin (TM) (2 μM), thapsigargin (TG) (1 μM) or 4-phenyl butyric acid (PBA) (500 μM) for 4 hours. (**A**) Cell lysates from LO2 cells were analyzed by immunoblotting with specific antibodies for the expression of the indicated molecules. The band density was quantified with ImageJ software. The quantitative data were obtained from three independent experiments, and are shown as the mean ± SEM. (**B**) Cell culture medium was collected and subjected to ELISA. Data are the mean ± SEM of three independent experiments. **C, D.** LO2 cells were transiently transfected with AT^WT^, AT^ΔINEL^ and AT^K241E^ plasmids for 48 hours and then treated with Eeyarestatin I (Eerl) (2 μM), MG-132 (1.2 μM), or E64d (10 μM) for 12 hours. (**C**) Cell lysates from LO2 cells were analyzed by immunoblotting with specific antibodies for the expression of the indicated molecules. The band density was quantified with ImageJ software. The quantitative data were obtained from three independent experiments, and are shown as the mean ± SEM. (**D**) Cell culture medium was collected and subjected to ELISA. A representative image of three experiments is shown, and the quantitative data are presented as the mean ± SEM of three experiments. Ctl, control. (*p < 0.05)

In response to ER stress, cells activate multiple adaptive mechanisms, known as the UPR, leading to ERAD [14]. In order to evaluate the involvement of ER stress in AT deficiency, we used tunicamycin (TM) and thapsigargin (TG) to induce ER stress in the transfected LO2 cells, and monitored the retention of AT. Enhanced ER stress promoted intracellular AT retention (Figure 3A) and reduced AT secretion (Figure 3B) in AT^ΔINEL^ cells, while there was no obvious effect on normal AT and AT^K241E^ cells (Figure 3A and 3B), highlighting that ER stress is essential for intracellular AT retention. We then inhibited ER stress with 4-phenyl butyric acid (PBA), and observed that inhibition of ER stress promoted AT^ΔINEL^ secretion (Figure 3A and 3B). Pretreatment of cells with the ERAD inhibitor Eeyarestatin I (Eerl), which directly binds to the p97 ATPase, an essential component of the ERAD machinery, further increased the intracellular retention of AT^ΔINEL^ and reduced AT^ΔINEL^ secretion (Figure 3C and 3D). Pretreatment with the proteasome inhibitor MG-132 produced similar results in terms of intracellular retention (Figure 3C and 3D), while lysosome inhibition (E64d) had only a trivial effect (Figure 3C and 3D), suggesting the importance of ERAD for intracellular AT degradation, likely through the proteasomal pathway. These data indicate that ER stress contributes to intracellular AT retention and degradation, while the inhibition of ER stress improves AT secretion.

## DISCUSSION

In this study, we identified one new small deletion within AT, which results in the loss of four amino acids (INEL) and is located at strand 3 of β-sheet A, a region highly conserved in *SERPINC1*. This mutation leads to type I AT deficiency by promoting the intracellular retention of AT, which induces ER stress. On the one hand, ER stress activates ERAD, which promotes AT degradation, while on the other hand, ER stress inhibits the synthesis of AT. Inhibition of ER stress partially restored AT levels, suggesting a novel potential way to alleviate AT deficiency.

AT is a serpin which is essential for hemostasis. Either quantitative (Type I) or qualitative (Type II) disorders lead to AT deficiency, breaking the elaborate equilibrium between hemostasis and thrombosis and usually resulting in pulmonary embolism and DVT [22, 23]. As AT deficiency is an autosomal dominant hereditary disorder, the most common genetic mutations causing type I AT deficiency are missense/nonsense point mutations, as well as small deletions/insertions which cause frameshifts within *SERPINC1* and thus prevent the expression of AT. Major gene deletions are rare, and homozygous deficiency is extremely rare and is associated with neonatal thrombosis, which is often fatal.

Type I AT deficiency can result from impaired translation due to unstable RNA or premature stop codons, as well as from protein misfolding/unfolding due to various mutations, which cause abnormal posttranslational modification and intracellular retention of AT or reduce its conformational stability [24, 25]. In our study, the deleted region was the product of exon 4, and manifested as a heterozygous mutation. Actually, another mutant within this region has been reported - K241E - which causes pleiotropic type II AT deficiency by inhibiting heparin binding [20]. Another mechanism for type I AT deficiency could be through causing glycosylation defects, but this has not yet been confirmed.

As type I AT deficiency may be pretranslational or posttranslational, we tested both possibilities in the present study. In cells transfected with the ΔINEL mutant, we found no difference in either mRNA levels or synthesized protein levels (assessed by Western blotting) compared with WT cells, while the mutant-transfected cells secreted much less AT, suggesting that there was a defect in intracellular retention. These results also revealed that posttranslational mechanisms cause the intracellular retention of AT, rather than mechanisms such as RNA instability or impaired translation. We also used the aforementioned K241E mutant AT as a control [20], but there were no obvious differences in AT synthesis and secretion in cells transfected with this mutant.

Intracellular retention is one of the fundamental mechanisms for type I AT deficiency [9, 23], resulting from conformational changes, misfolding, etc. We used computer simulation to determine whether there was a conformational change in the mutated AT (see Supplemental figure), but found no obvious difference between the mutated and WT forms. Therefore, we inferred that misfolding might be one of the reasons for intracellular retention of AT. However, misfolded or unfolded proteins, especially secreted proteins, induce ER stress. When we measured the “sensors” for ER stress, we did find substantial increases in the expression of GPR78, p-IRE1 and p-PERK and the cleavage of ATF6, implying that ΔINEL endogenously promotes ER stress.

However, it was not clear whether ER stress was the cause or effect of AT intracellular retention, so we took further steps to resolve this question. Promoting ER stress with agonists reduced the secretion of AT, while inhibition of ER stress instigated AT secretion, demonstrating that ER stress promotes the intracellular retention of AT. In compensation, ER stress initiates ERAD or autophagy to degrade proteins [15, 17, 26, 27]. Surprisingly, when we treated the ΔINEL cells with an ERAD inhibitor, the secretion of AT was further inhibited, while there were no observable effects on WT and K241E AT secretion, illustrating that ER stress is essential for inhibiting ΔINEL secretion. In addition, proteasomal inhibition produced a similar result to the ERAD blockade, while lysosomal inhibition had little effect.

Overall, our study identified a new mutation (INEL deletion) causing type I AT deficiency, and uncovered a novel mechanism for AT retention through enhanced ER stress. These findings imply that AT deficiency due to AT retention may be treated by reducing ER stress, and suggest that alternative methods of alleviating or treating AT deficiency are possible. Certainly, further study is needed to reveal how ER stress is involved in AT retention; for example, do different signaling cascades (IRE1, PERK, or ATF6) for ER stress have differential activities in the regulation of AT retention? Is ER stress a general regulator causing the intracellular retention of other mutant forms of AT, or just the ΔINEL mutant? Therefore, additional research is required to support our results.

## MATERIALS AND METHODS

### Pedigree research and phenotype tests on the proband and relatives

The proband was referred to our hospital due to a headache and vomiting in 2013, and was diagnosed as having cerebral venous thrombosis after clinical and imaging approaches. All family members were interviewed for their medical history, and a detailed family history was obtained from the patient and her family members, with a particular emphasis on the occurrence of prior thrombosis events. Venous blood samples were collected from the patient and family members, with written consent and formal approval from all involved persons. This study was approved by the Ethics Committee of Shanghai Ninth People's Hospital, Shanghai Jiao Tong University School of Medicine.

AT heparin cofactor activity (AT:A) was quantified through a chromogenic substrate method involving inactivation of Factor Xa in the presence of excess of heparin (Instrumentation Laboratory), while AT antigen (AT:Ag) was quantified by an immunoturbidimetry method using the Beckman AT reagent in conjunction with a Beckman IMMAGE® 800 Immunochemistry System and Calibrator 2 (Beckman Coulter, CA, USA). The activated partial thromboplastin time, prothrombin time and international normalized ratio were evaluated, and the thrombodynamics test, D-dimer (product of thrombin degradation) test and fibrinogen testing were also conducted.

### Genomic DNA extraction, PCR, DNA and DNA sequencing

Genomic DNA was extracted from peripheral blood leukocytes by standard methods. Briefly, genomic DNA libraries were prepared according to the protocols recommended by Illumina. Whole-exome enrichment was performed with a TruSeq Exome Enrichment kit (Illumina, San Diego, CA). Captured DNA libraries were sequenced with an Illumina HiSeq 2500 Genome Analyzer, yielding 200 (2 × 100) base pairs from the final library fragments.

The mutation was named according to the standard international nomenclature guidelines recommended by the Human Genome Variation Society (http://www.hgvs.org/varnomen). The mRNA (GenBank: NM_000488.3) and protein (GenBank: P01008) sequences of *SERPINC1* were used as the reference sequences.

### Reagents and antibodies

Tunicamycin (TM), thapsigargin (TG), lysosome inhibitor E64d, and proteasome inhibitor MG-132 were purchased from Calbiochem (Billerica, MA). Cycloheximide was obtained from Sigma (St. Louis, MO). Antibodies against c-Myc, AT, GRP78, p-IRE1, p-PERK, and ATF6 were obtained from Cell Signaling Technology (Boston, MA). ELISA Kits for detecting AT were purchased from R&D Systems (Minneapolis, MN). HRP-conjugated anti-mouse and anti-rabbit secondary antibodies were purchased from Jackson ImmunoResearch (West Grove, PA).

### Cell culture and transfection

LO2 cells are derived and immortalized from human embryonic liver, and have been described as normal hepatocytes [28]. The LO2 cell line was from the Key Laboratory of Molecular Virology and Immunology, Institute Pasteur of Shanghai, Chinese Academy of Sciences (Shanghai, China).

LO2 and HEK293 cells were cultured in DMEM supplemented with 10% FBS, 100 U/mL penicillin, and 100 μg/mL streptomycin (Invitrogen) at 37°C in a humidified chamber with 5% CO_2_.

LO2 and HEK293 cells were plated in 24-well plates (2 × 10^5^ cells/well) and transfected the next day with plasmids and lipofectamine 3000 according to the manufacturer's instructions. After 48 hours, the cells were analyzed or used for other experiments.

### Recombinant plasmid construction of WT and mutant AT

Full-length *SERPINC1* was PCR-amplified from first-strand cDNA libraries, prepared from poly(A)^+^ mRNA isolated from human placenta, and was subcloned into a PRK5 mammalian expression vector containing an N-terminal Myc epitope tag (AT WT). cDNAs for *AT* mutants (AT^K241E^ and AT^ΔINEL^) were generated by PCR amplification with the full-length *AT* cDNA as the template, and were constructed with a Quikchange XL Site-Directed Mutagenesis Kit (Agilent, Santa Clara, CA).

### Immunoblotting

Immunoblotting was performed as previously described [29]. Briefly, cells were collected and cell lysates were prepared with lysis buffer (1% Nonidet P-40, 50 mM Tris-HCl, pH 7.4, 250 mM NaCl, with protease inhibitor cocktail [Roche Applied Science]). Cell lysates were subjected to 10% sodium dodecyl sulfate-polyacrylamide gel electrophoresis for about 2 hours and then were transferred to polyvinylidene fluoride membranes. After 1 hour of blocking in 5% fat-free milk at room temperature, the membranes were incubated with the primary antibody and then with the proper HRP-conjugated secondary antibody. The immunoreactive bands were detected with ECL plus immunoblotting detection reagents (GE Healthcare). For some immunoblots, the band density was quantified with ImageJ software (National Institutes of Health).

### RT-PCR

The specific primers for *SERPINC1* amplification were: Forward 5′–gttgc ccttc aaagg tgatg a-3′, Reverse 5′-cagat cgaca aggcc catgt c-3′. Total RNA was extracted from cells with TRIzol^®^ reagent (Invitrogen, Carlsbad, CA). Then, the RNA integrity was confirmed by electrophoresis and the concentration was determined. RNA was reverse-transcribed with Superscript II (Invitrogen) and random hexamer primer, and quantitative PCR was performed with a FastStart Universal SYBR Green Master Kit (Roche) and an ABI PRISM 7900HT system (Applied Biosystems). The reaction protocol was set as 95 °C for 5 min and 35 cycles of 95°C for 15 s, 60°C for 60 s, and 72°C for 5 min. The expression of genes of interest was normalized to that of the reference gene (β-actin), and expression was calculated with the 2^−ΔΔCt^ method.

### ELISA

Culture medium was collected after centrifugation to remove cell debris, and was stored at -80°C until analysis. AT was detected with an ELISA kit (R&D Systems, Minneapolis, MN) according to the manufacturer's instructions.

### Cell viability assay

Cell viability was assessed with a Vybrant® MTT Cell Proliferation Assay Kit according to the manufacturer's instructions (Invitrogen, Carlsbad, CA). LO2 cells were plated in 96-well plates at a density of 5×10^2^ cells per well and transfected with a plasmid containing WT or mutant *AT*. The cell viability was assessed at various time-periods (0, 1, 2, 3, 4 d) after transfection. The absorbance of each well was measured at 570 nm with an ELISA reader (Biotek, Winooski, VT, USA).

### Statistical analysis

A two-tailed Student's *t* test or one-way ANOVA was used for statistical analyses in this study. A *p* value less than 0.05 was considered to be statistically significant.

## SUPPLEMENTARY MATERIALS FIGURE



## Abbreviations

AT: antithrombin
DVT: deep vein thrombosis
ER stress: endoplasmic reticulum stress
ERAD: endoplasmic reticulum-associated degradation
IRE1: inositol-requiring kinase 1
PERK: double-stranded RNA-activated protein kinase (PKR)-like endoplasmic reticulum kinase
SERPIN: serine protease inhibitor
