# Supplementary material for: A small deletion in SERPINC1 causes type I antithrombin deficiency by promoting endoplasmic reticulum stress
Source: Oncotarget. 2016 Sep 30;7(47):76882–90. doi: 10.18632/oncotarget.12349 (PMC5363556; doi:10.18632/oncotarget.12349)
Supplement: Supplementary file 1 [file oncotarget-07-76882-s001.pdf]

A small deletion in *SERPINC1* causes type I antithrombin deficiency by promoting endoplasmic reticulum stress

SUPPLEMENTARY FIGURE

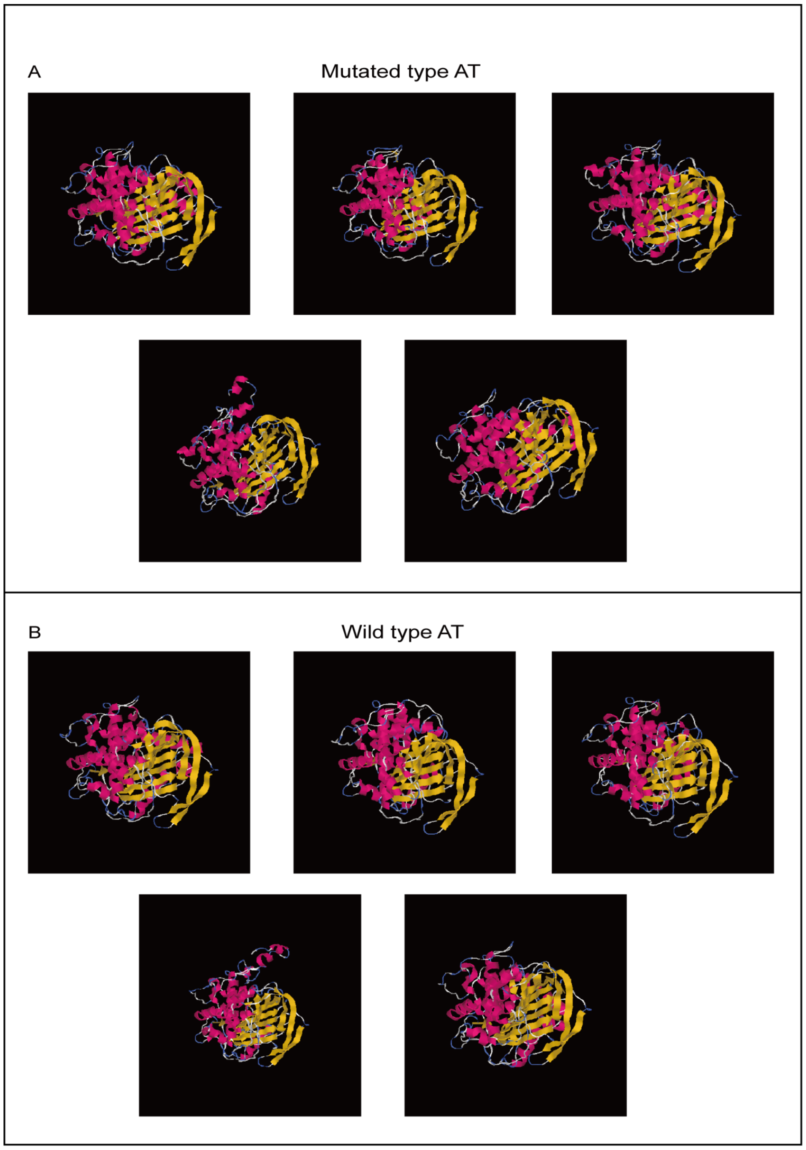

Supplementary Figure S1: Conformation of mutated and wild-type AT in a computer simulation. A. Conformation of mutated AT. **B.** Conformation of wild-type AT.
